# Changes of spino-pelvic characteristics post-THA are independent of surgical approach: a prospective study

**DOI:** 10.1007/s00402-024-05739-y

**Published:** 2025-02-17

**Authors:** Moritz Wagner, Jeroen Verhaegen, Camille Vorimore, Moritz Innmann, Ottawa Arthroplasty Group, George Grammatopoulos

**Affiliations:** 1 Medical University Innsbruck, Innsbruck, Austria; 2 The Ottawa Hospital - General Campus, Ottawa, Canada; 3 University Hospital Antwerp, Edegem, Belgium; 4 Heidelberg University Hospital, Heidelberg, Germany; 5 Paracelsus Medical University, Salzburg, Austria

**Keywords:** Pelvic tilt, Outcomes, Approach, Change, Spinopelvic

## Abstract

**Aims:**

Spinopelvic characteristics change after THA. Whether this change varies between approaches, is of interest for pre-op cup orientation planning. The aims of this study were to (1) Characterize changes in standing PT amongst patients with hip osteoarthritis treated with THA; (2) Test whether certain patient-related factors may predict PT change; and (3) Assess the association between surgical approach and PT change.

**Methods:**

This was a prospective, two-center, radiographic outcome study including 424 hips, consisting of anterior approach for 171 (40.3%) hips, lateral approach for 181 hips (42.7%) and posterior approach for 72 hips (17.0%). Spinopelvic characteristics were determined from lateral radiographs (before and one year after THA). Parameters of interest included: Pelvic tilt (PT), lumbar lordosis, sacral slope, pelvic incidence. PT change of more than 7 ° was considered clinically relevant.

**Results:**

Pelvic tilt increased by 2 ° from 15.1 ° (± 8.9) to 17.1 ° (± 9.7) after THA (p < 0.001). 19 hips (4.5%) experienced a relevant PT decrease, 337 (79.5%) had no clinically significant change in pelvic tilt, and 68 (16.0%) showed a moderate increase. Age, female sex and preoperative spinopelvic parameters including PT, SS and PI were predictive of PT change more than 7 °. PT increased most with lateral approach (2.9 ± 6.2) and least with anterior approach (1.1 ± 6.2, p = 0.024).

**Conclusion:**

Preoperative PT is the best predictor for PT change. PT is generally normalizing after THA and patients with low PT due to hip flexion contractures tend to increase PT after THA, few patients with high PT will decrease after THA. Anterior approach with capsulectomy was associated with the least change in PT post-THA. However, the approach-specific changes, although statistically significant, were too small to be considered during clinical practice, therefore no approach-specific prediction of PT change needs to be considered during preoperative planning for primary THA.

## Introduction

Acetabular component (cup) orientation is important for outcome following total hip arthroplasty (THA) [1–3]. Over the last decade, there has been heightened interest towards the identification of optimal, functional cup orientation, thus moving away from the static, universal, ‘Safe-Zone’ [2, 4, 5]. This paradigm shift has been promoted by the observation that dislocations may occur with cups within the traditional “Safe-Zone” [4, 5]. The study of functional cup orientation relies on the characterization of individual sagittal spinopelvic characteristics, thus determining how the pelvis moves in space in harmony with the lumbar spine and femur [2, 6].

Functional acetabular cup orientation considers changes in functional pelvic position, primarily pelvic tilt (PT) [2, 7]. On average, 13° increase in PT is associated with 10 ° increase in cup version [8]. Standing PT of volunteers and patients with hip arthritis varies widely (14 ° ± 8 °), with values being dependent upon age, sex, degree of spinal degeneration/fusion and spinal muscle atrophy [6, 9, 10]. Spinopelvic abnormalities caused by hip flexion contracture (osteoarthritis) generally “normalize” after THA [11–14]. Predicting PT change post-THA can help with pre-operative planning of cup orientation to achieve individual, functional, target.

Although it has been established that spinopelvic characteristics change after THA [15–17], the effect of surgical approach on changes in these characteristics post-THA is unknown. For example, it could be hypothesized that approaches with anterior capsulectomy are associated with greater relief of hip flexion contractures leading to a greater increase in PT post-operatively. This would be associated with greater functional cup anteversion, which may in turn lead to functional cup mal-orientation and increased risk of anterior dislocation.

The aims of this study were to (1) Characterize changes in standing PT amongst patients with hip osteoarthritis treated with THA; (2) Test whether certain patient-related factors may predict PT change; and (3) Assess the association between surgical approach and PT change.

## Patients and methods

This was an international, prospective, clinical, study with two participating centers, Center A (University of Ottawa's Division of Orthopaedic Surgery, Ottawa, Canada) and Center B (Heidelberg University Hospital, Heidelberg, Germany). Institutional review board approval was obtained for both tertiary academic institutions (OHSN-REB 20200597-01H; IRB S‐065/2017) and this study conformed with the Declaration of Helsinki from 2008 in all aspects. Patients between age 40 and 85 years with end-stage osteoarthritis of the hip awaiting THA were included. Exclusion criteria were lack of sufficient quality radiographs before and minimum one year after surgery to determine comprehensive spinopelvic measurements as detailed below.

The sample size for the study was determined using an a-priori power analysis based on fixed effects omnibus one-way analysis of variance (ANOVA) to compare PT between approaches. Power analysis (G*Power, Heinrich Heine Universität Düsseldorf; Düsseldorf, Germany) was based on one-way omnibus ANOVA with three arms (anterior, lateral and posterior approach), effect size of 0.2, α of 0.05, and power (1 – β) of 0.95 resulting in a minimum total sample size of 390 patients [18].

### Study cohorts

The cohort from Center A initially consisted of 633 hips, treated by 9 different surgeons with mixed (anterior, lateral and posterior) surgical approaches. 361 hips were excluded for lacking/ not having reached follow-up at one year. This left 272 complete cases. The cohort from Center B initially comprised 250 hips, all of whom underwent a lateral approach. Exclusions in this cohort consisted of 62 hips that lacked radiographic follow-up at one year, resulting in 188 complete cases. To reduce the risk of bias, patients, as per approach, were matched for BMI, age and sex leading to a final study population of 424 patients which were used for all further analyses. The mean age was 65 (± 10) years-old, 222/424 (52%) were female and mean BMI was 28 (± 5) kg/m^2^. 171 (40%) patients underwent THA through anterior-, 181 (43%) lateral- and 72 (17%) through posterior-approach.

### Surgical approaches

THA was performed (a) supine using the anterior approach (direct anterior approach; Hueter; modified Smith Peterson, with and without positioning table) for 168 (39.6%) hips, (b) with lateral approach (direct lateral; Hardinge, in lateral decubitus position at Center A and in supine position at Center B) for 181 hips (42.7%) and using (c) posterior approach (posterolateral; Moore) for 72 hips (17.0%). Patients were grouped according to their surgical approach.

### Capsular management

Patients were also grouped according to capsular management to investigate the effects of capsulectomy versus capsulotomy with consequent capsular repair. Capsulotomy with repair was performed for all posterior approaches (72 hips, 17%). Anterior (partial) capsulectomy was performed for all direct lateral approaches (181 hips (42.7%). Both techniques, capsulectomy (58 hips, 13.7%) and capsulotomy with repair (110 hips, 25.9%) were performed for anterior approaches, depending on surgeon preference. Proximal femoral capsular and soft tissue release varied between cases depending on necessary exposure.

### Radiographic evaluation

**Fig. 1:**
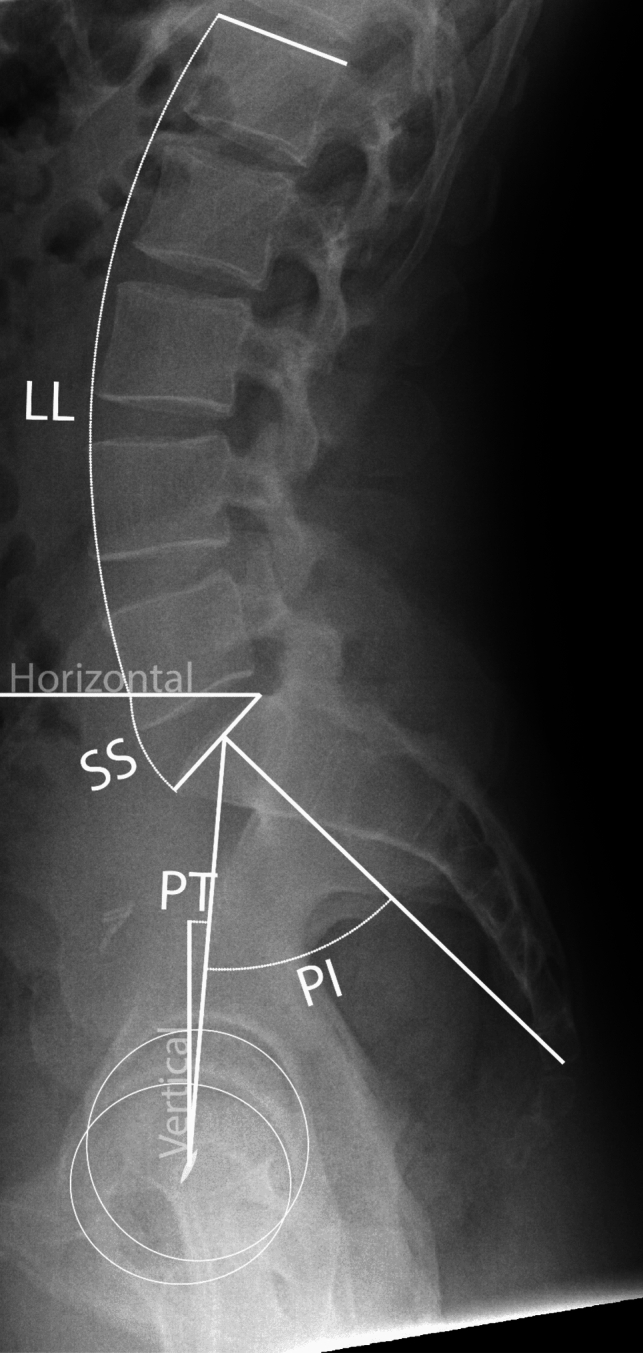
Radiographic outcome measures of five spinopelvic parameters from standing lateral pelvic radiographs before and one year after total hip arthroplasty (THA). *PT* pelvic tilt, *PI* pelvic incidence, *SS* sacral slope, *LL* lumbar lordosis

Spinopelvic characteristics were determined from standing lateral pelvic radiographs. Those were obtained before and one year after THA. Radiographs were checked for sufficient quality, including accurate projection and visualization of the complete lumbar spine including L1, complete pelvis and proximal femur to allow for measurement of complete spinopelvic parameters. Those included lumbar lordosis (LL), sacral slope (SS), pelvic incidence (PI), and PT. An example of the measurements for spinopelvic parameters has been depicted in Fig. 1. All radiographic measurements were conducted by a single reviewer before and one year after surgery using digital imaging analysis software (SterEOS; EOS Imaging Inc, Paris, France; WebPACS Lite; Change Healthcare, Nashville, USA). To assess reliability, measurements were repeated two weeks later on 10% of randomly selected data sets by two blinded reviewers. Intraobserver and interobserver reliability were evaluated using average measure correlation coefficients calculated with a two-way random effects model for absolute agreement, demonstrating excellent reliability (range: 0.97; 95% CI 0.88–0.99 to 1.0; 95% CI 0.99–1.00)[19]. Preoperative radiographic measurements have been summarized with demographic parameters in Table 1.

**Table 1:** Age, BMI, surgical parameters and preoperative spinopelvic measurements for` Center A and Center B

	Center A	Center B	Overall	P-Value
Sex (female)	136 (54.2%)	86 (49.7%)	222 (52.4%)	0.210
Age	65.3 ± 9.5	65.8 ± 10.3	65.5 ± 9.8	0.587
BMI	28.1 ± 4.9	28.0 ± 4.9	28.0 ± 4.9	0.791
Anterior approach	171 (68.1%)	0 (0%)	171 (40.3%)	< 0.001
Lateral approach	8 (3.2%)	173 (100%)	181 (42.7%)	< 0.001
Posterior approach	72 (28.7%)	0 (0%)	72 (17.0%)	< 0.001
Capsule Repair	182 (73.4%)	0 (0%)	182 (43.2%)	< 0.001
Capsulectomy	66 (26.6%)	173 (100%)	239 (56.8%)	< 0.001
Preoperative PT	16.0 ± 8.8	13.8 ± 8.8	15.1 ± 8.9	0.009
Preoperative LL	54.0 ± 13.0	53.6 ± 13.1	53.9 ± 13.0	0.716
Preoperative SS	38.4 ± 9.3	42.7 ± 9.6	40.1 ± 9.6	< 0.001
Preoperative PI	54.5 ± 10.8	56.6 ± 12.8	55.4 ± 11.7	0.062

*BMI*: Body Mass Index (m^2^/kg)

### Outcome measures

The primary outcome measure was the change in PT after THA. PT change of more than 7 ° leading to 5 ° change of cup anteversion was considered clinically relevant and described as *moderate increase/decrease*; a PT change of more than ± 13 ° leading to ± 10 ° of acetabular cup anteversion was considered a high increase/decrease [8]. Secondary outcome measures included the change in all the other spinopelvic parameters.

All outcome measures were compared before and one year following THA. Changes were assessed (1) for the overall cohort, (2) considering patient factors, including preoperative spinopelvic factors, demographic factors, BMI and (3) as per surgical approaches, including capsular management.

### Data analyses

Analyses were performed with SPSS (Version 29, IBM; NY, USA); a p-value less than 0.05 was considered significant. Descriptive statistics, presented as means and standard deviations (SDs), were utilized to summarize pre- and postoperative spinopelvic parameters. Comparisons between pre- and postoperative parameters were made with a two-sided paired t-test.

Univariate analyses were performed to test which patient- and surgical- factors were associated with greater change in PT. Factors with significant change and those that showed a possible trend (p < 0.1) were included in the linear regression analysis evaluating the influence on predictors of spinopelvic change.

Kolmogorov–Smirnov test confirmed normal distribution, ANOVA was performed to compare means between groups (anterior-, lateral and posterior approach). Both absolute postoperative values and relative changes from before to after THA have been subjected to this analysis.

## Results

1. Overall changes in standing PT after THA

Spinopelvic measurements are summarized in Table 1. Pelvic tilt increased from 15.1° (± 8.9) to 17.1° (± 9.7), showing a relative change of 2.0° (± 6.3) (p < 0.001). Among the 424 patients, 19 (4.5%) experienced a moderate PT decrease, 337 (79.5%) had no clinically significant change in pelvic tilt, and 68 (16.0%) showed a moderate increase. Eight patients (1.9%) exhibited a high *decrease* in pelvic tilt and 13 (3.1%) experienced a high *increase* in pelvic tilt. Lumbar lordosis increased from 53.9° (± 13) to 54.9° (± 13.8), with a relative change of 1° (± 0.8).

(2) Patient factors predictive of PT change

**Fig. 2:**
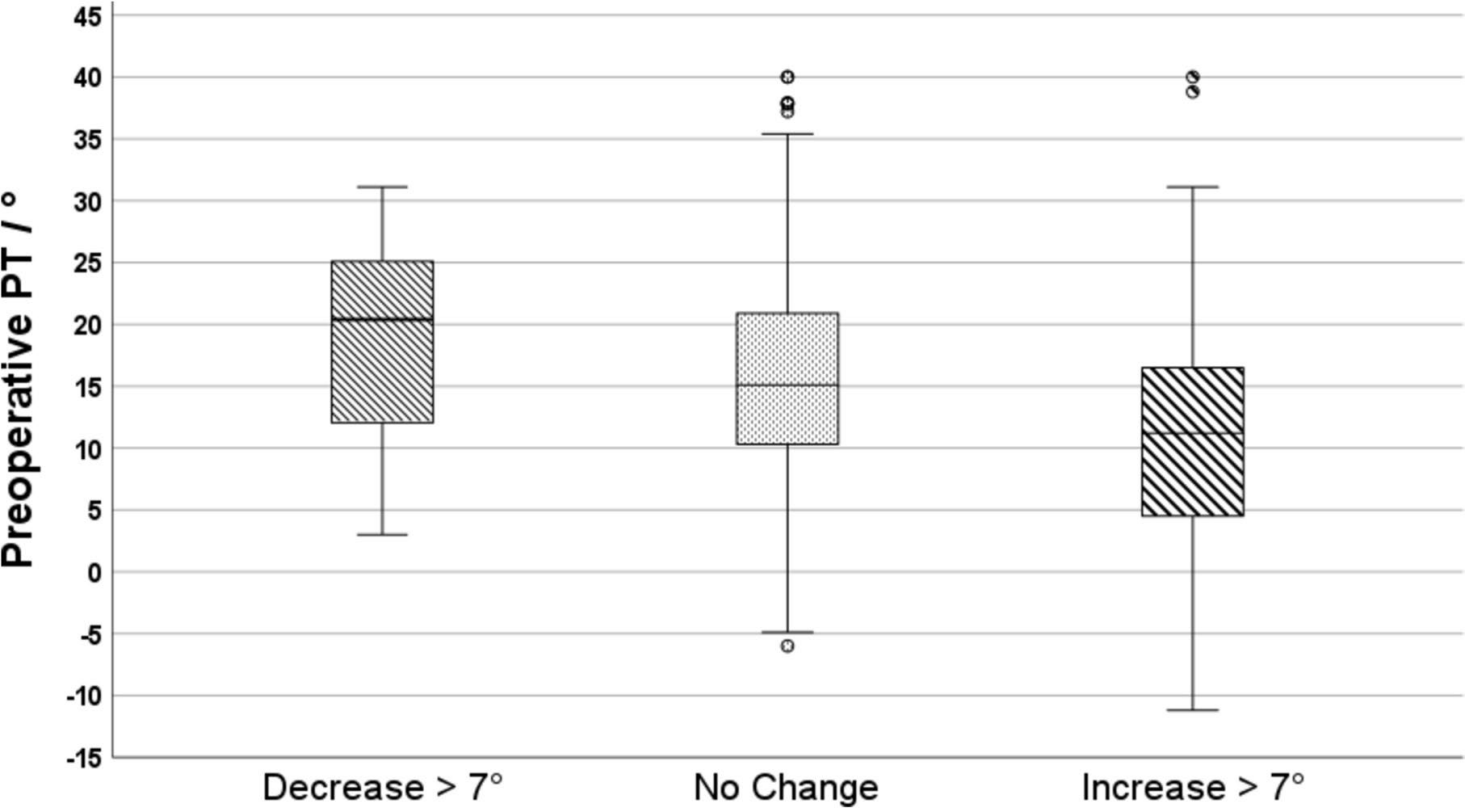
Box-plot showing changes in PT after THA depending on the preoperative PT. *PT* pelvic tilt, *THA* total hip arthroplasty

Age, female sex and preoperative spinopelvic parameters including PT, SS and PI were predictive of PT change more than 7 °. Those were included in the multivariate linear regression analysis. The model fit was weak with an R^2^ of 0.182 (p = 0.001). Unstandardized regression coefficients (B), confidence intervals and levels of significance for predictive parameters were summarized in Table 2. Patients with higher preoperative pelvic tilt tended to decrease pelvic tilt, likewise, patients with lower preoperative pelvic tilt tended to increase pelvic tilt after THA (Fig. 2).

(3) PT change between surgical approaches

**Table 2:** Multivariate linear regression analysis of predictors for PT change with the unstandardized regression coefficient, level of significance and confidence intervals

	B (Coefficient)	95% CI lower bound	95% CI upper bound	P-Value
Age	0.094	0.035	0.153	0.002
Female Sex	−1.824	−2.938	−0.71	0.001
Preoperative PT	−0.433	−0.527	−0.338	< 0.001
Preoperative LL	−0.167	−0.228	−0.106	< 0.001
Preoperative PI	0.284	0.203	0.365	< 0.001

**Fig. 3:**
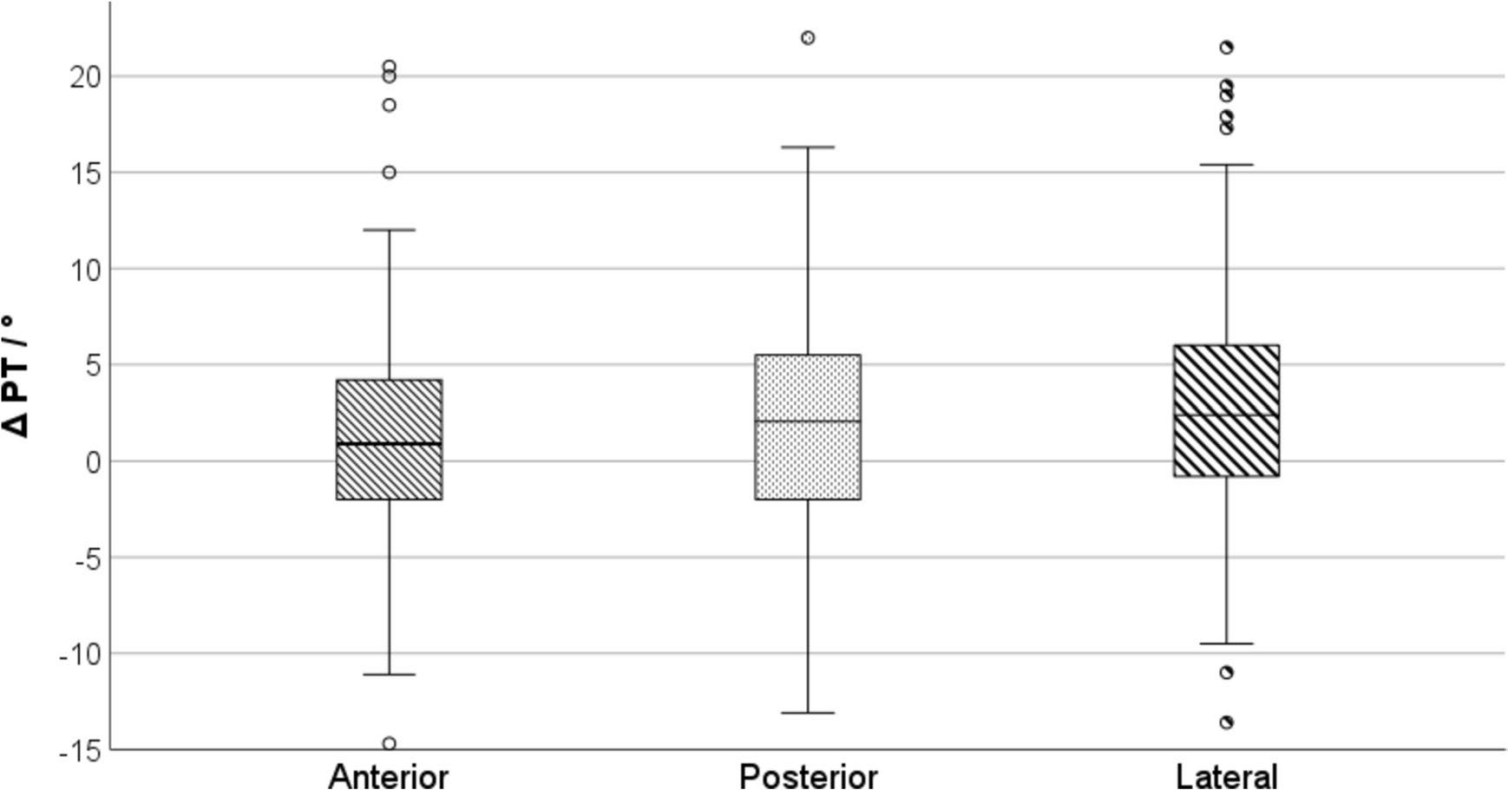
Box-plot showing relative changes in PT after THA depending on surgical approach used. *ΔPT* change in pelvic tilt, *THA* total hip arthroplasty

All demographic and spinopelvic parameters were compared between surgical approaches with univariate analysis (Table 3). PT increased most with lateral approach (2.9 ± 6.2) and least with anterior approach (1.1 ± 6.2, p = 0.024). Within the anterior approach, there were significant differences in PT change between capsulotomy with repair (1.6 ± 6.2) and capsulectomy (0.3 ± 5.4; p = 0.016). The magnitude of approach dependent PT change has been summarized in Fig. 3.

**Table 3:** Univariate analysis of demographic parameters and spinopelvic parameters before THA, one year after THA and changes dependent on surgical approach

		Anterior approach	Lateral approach	Posterior approach	Total	P-Value
Demographic	Sex (female)	n = 88 (51.5%)	n = 92 (50.8%)	n = 42 (58.3%)	n = 222 (52.4%)	0.535
	Age	64.5 (± 0)	66 (± 0)	67 (± 0)	65.5 (± 0)	0.075
	BMI	27.6 (± 0)	28.1 (± 0)	29 (± 0)	28.1 (± 0)	0.063
Preoperative	PT	16 (± 8.7)	13.8 (± 9)	16.4 (± 8.6)	15.1 (± 8.9)	0.031
	LL	54.6 (± 13.3)	53.4 (± 13)	53.2 (± 12.4)	53.9 (± 13)	0.644
	SS	38.5 (± 9.3)	42.3 (± 9.8)	38.5 (± 8.8)	40.1 (± 9.6)	< 0.001
	PI	54.6 (± 11.1)	56.3 (± 12.7)	54.8 (± 10.3)	55.4 (± 11.7)	0.424
Postoperative	PT	17.1 (± 9.6)	16.7 (± 9.8)	18 (± 10.1)	17.1 (± 9.7)	0.463
	LL	54.9 (± 12.2)	54.5 (± 13.1)	55.7 (± 12.9)	54.9 (± 12.3)	0.865
	SS	37.3 (± 9.6)	39.1 (± 9.4)	38.8 (± 9.4)	38.3 (± 9.5)	0.204
	PI	54.3 (± 12)	55.9 (± 12.8)	56.8 (± 11.5)	55.4 (± 12.3)	0.230
Δ	PT	1.1 (± 6.2)	2.9 (± 6.2)	1.6 (± 6.8)	2 (± 6.3)	0.035
	LL	0.3 (± 6.3)	1.1 (± 3.9)	2.5 (± 7.1)	1.0 (± 6.1)	0.268
	SS	−1.3 (± 6.5)	−3.2 (± 6.6)	0.2 (± 7.3)	−1.8 (± 6.8)	< 0.001
	PI	−0.3 (± 6.7)	−0.4 (± 6.1)	2 (± 8.4)	0.1 (± 6.8)	0.030

*THA*: Total hip arthroplasty

## Discussion

**Fig. 4:**
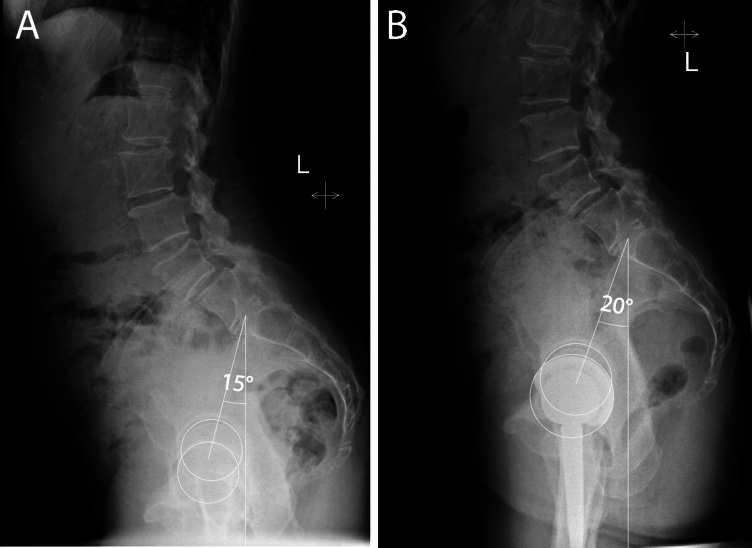
Patients with lower preoperative PT tended (A) tended to increase PT after THA, example of a patient with a slight clinically non-significant PT increase of 5°. *PT* pelvic tilt, *THA* total hip arthroplasty

Following total hip arthroplasty, pelvic tilt increased only minimally (2 ± 6°). Although the mean change was small, one-in-five patients (21%) changed their standing PT post-THA by more than 7 °. The majority of these increased PT (16%). The most predictive factor for PT change was preoperative PT; higher pre-operative PT was associated with decrease in PT post-operatively, whilst a lower PT pre-operatively was associated with an increase in PT (Fig. 4) [16]. Although statistically significant, the effect of surgical approach has likely only a small, if any, clinical relevance. Contrary to our hypothesis, anterior approach, especially in cases with capsulectomy, was associated with the least change in PT. It is plausible that the scarring of the anterior structures following capsulectomy prevents the expected increase in PT that would take place following resolution of the fixed-flexion contracture. This persistent low PT (hip flexion contracture) may be due to irritation of the iliopsoas tendon and could be counteracted by capsular repair. Our results demonstrated more PT increase with capsular repair (1.6° vs 0.2°, p = 0.016). Overall, changes between surgical approaches were too small to be considered during clinical practice. These results are reassuring that no approach-related considerations need to take place as part of the preoperative surgical planning.

### Overall PT changes after THA

Changes in PT reported in this cohort are in accordance with previous literature. Innmann et al. found 13.7° standing PT before and 15.3 ° PT after THA (increase of 1.6 °) [16]. Heckmann et al. recently reported an increase of 7.4° (± 4.5) [14, 20] from preoperative supine to postoperative standing anterior pelvic plane tilt (APPT), using a posterior approach. Similarly, Katsura et al. (2022) reported a change of 5.0° (± 4.0) in APPT at one year follow-up, without specifying surgical approach [13]. Our study has important differences to the aforementioned studies. In Heckmann et al. (2024), the high changes in PT before and after surgery were only seen when comparing preoperative supine and postoperative standing radiographs. Most of that PT change is likely associated with a difference in posture, rather than from the changes following THA [21]. Even when adjusting for the known PT increase from supine to standing, which is reported to be + 4° (± 4°)[22], the results from Heckmann et al. (2024) are slightly higher than the PT change we found for the posterior approach. Tang et al. (2024) reported on pre- and post-THA standing pelvic tilt in 143 patients using full body standing lateral biplanar radiographs (EOS). The authors found a mean change in PT of + 5° (± 4.1°) [23], which is greater than this cohort’s findings, and most likely due to the high number of patients with global sagittal imbalance. Patients with global sagittal imbalance accounted for 26% of their cohort and changed the PT by an average of 6.6°[23].

### Predictors for PT change

Several morphological (Preoperative PT, SS and LL) and demographic (female sex and age over 75) were associated with PT change. However, prediction of PT remains complex, and the linear regression model from this study explains only little variability in the data. The best predictor in this cohort was pre-operative PT. Patient with lower PT tended to increase after THA and vice-versa, patients with a normal PT (10–20°) had little changes. Few studies have investigated predictors for PT change. Pour et al. (2023) found standing SS most predictive for PT change [24]. Masanori et al. (2021) found reduction in PT for patients with low BMI intraoperatively measured with accelerometer-based navigation system, those effects normalized at one year after surgery [25]. Fujii et al*.* (2023) used machine learning algorithms to predict one year change in PT and they identified patients with preoperative BMI > 25.4 and PT > 15.5 as being at risk for significant PT change [12]. Tang et al. reported on a reliable prediction model for PT change [23]. This method relies on standing radiographs, involving the whole spine, to determine the spinal vertical axis (SVA), thus associated with significant radiation and dependency on EOS, reducing its practical utility in most arthroplasty practices. Further large-scale prospective data is required to determine better models that predict post-operative change.

### Influence of surgical approach on PT change

Hip osteoarthritis is a known cause of hip flexion contractures, balanced by a forward leaning posture leading to a decrease PT. Therefore, it seems plausible that, by restoring joint function through THA leading to full hip range of motion, compensatory low pelvic tilt can normalize and increase. In this study PT increased from 15.1 (± 8.9) with hip osteoarthritis to 17.1 (± 9.7) after THA. Relative to the effect of alleviating the fixed-flexion contractures associated with a THA, the effect of the approach was small. A previous study compared PT changes after THA for two surgical approaches on a smaller sample size without differences [26]. Our study with higher sample size including three most commonly used approaches found differences in PT change (Fig. 2), which give reason to reflect on underlying causes. However, relative changes were too small to be worth considering during day-to-day clinical practice, when considering that at least 7° of PT increase is required to result in 5° of added functional cup anteversion.

We generally found slightly greater PT increase for the lateral, posterior, and anterior approach with capsular repair, in comparison to anterior approach with capsulectomy. It is plausible that an anterior capsulectomy would lead to scarring of the anterior structures (iliopsoas, ilio-capsularis and capsular remnants/neo-capsule). Studies in patients with peri-acetabular osteotomies have illustrated the gradual increase in PT in the postoperative period [27], further supporting the notion that alleviation of flexion contracture and associated stretching of psoas leads to increase PT in post hip surgery. Future, larger prospective cohort studies would be able to study the effect of capsulectomy in greater detail. Nevertheless, the results of this study regarding the anterior approach are in contrast with the conclusions of Heckmann et al., who found that patients post-THA with the anterior approach are at increased risk of high functional anteversion due to the changes seen post-THA. We found little overall change after THA and clinically non-significant PT change depending on surgical approach, thus no approach specific cup orientation adjustments are necessary.

Limitations of this study include the lack of assessment of coronal reconstruction (implant position, leg length and offset). Therefore, it can’t be ruled out that other factors, especially subtle changes in bony biomechanical properties associated with those approaches, attributed to PT change, or whether the changes seen were solely related to the soft tissues. We only assessed a single position (standing) and did not perform a dynamic assessment with seated radiographs. This study design with isolated standing radiographic outcome measures was chosen to increase the applicability of our results for decision making given that a standing lateral pelvic radiograph is a universal form of assessment included in protocols of hip-spine analysis, regardless whether the deep- or relaxed seated assessments are performed, which demonstrate differences between them [2].

The follow-up period of this study was one year. We assume that there would be no further changes in spinopelvic alignment past that time period, however long-term follow-up studies are necessary as studies to-date have reported on long-term changes with supine assessments [28]. This study was performed with two participating institutions. The theoretical disadvantage of multiple institutions may be systematic error in outcome measures; however outcome measures have been checked for plausibility and intra- and interobserver reliability by fellowship trained surgeon (M.I.) participating in outcome assessment from both institutions. Both institutions had varying proportions of surgical approaches used, introducing possible selection bias.

## Conclusion

PT increases slightly (2°) at one year after THA. Preoperative PT is the best predictor for PT change. PT is generally normalizing after THA and patients with low PT due to hip flexion contractures tend to increase PT after THA, few patients with high PT will decrease after THA. Anterior approach with capsulectomy was associated with the least change in PT post-THA. However, the approach-specific changes, although statistically significant, were too small to be considered during clinical practice, therefore no approach-specific prediction of PT change needs to be considered during preoperative planning for primary THA.

## Data Availability

No datasets were generated or analysed during the current study.
